# *P. gingivalis*-LPS Induces Mitochondrial Dysfunction Mediated by Neuroinflammation through Oxidative Stress

**DOI:** 10.3390/ijms24020950

**Published:** 2023-01-04

**Authors:** Ambika Verma, Gohar Azhar, Xiaomin Zhang, Pankaj Patyal, Grishma Kc, Shakshi Sharma, Yingni Che, Jeanne Y. Wei

**Affiliations:** Donald W. Reynolds Department of Geriatrics and Institute on Aging, University of Arkansas for Medical Sciences, Little Rock, AR 72205, USA

**Keywords:** *P. gingivalis*-LPS, neuroinflammation, oxidative stress, mitochondrial dysfunction

## Abstract

*Porphyromonas gingivalis (P. gingivalis),* a key pathogen in periodontitis, is associated with neuroinflammation. Periodontal disease increases with age; 70.1% of adults 65 years and older have periodontal problems. However, the *P. gingivalis*- lipopolysaccharide (LPS)induced mitochondrial dysfunction in neurodegenerative diseases remains elusive. In this study, we investigated the possible role of *P. gingivalis*-LPS in mitochondrial dysfunction during neurodegeneration. We found that *P. gingivalis*-LPS treatment activated toll-like receptor (TLR) 4 signaling and upregulated the expression of Alzheimer’s disease-related dementia and neuroinflammatory markers. Furthermore, the LPS treatment significantly exacerbated the production of reactive oxygen species and reduced the mitochondrial membrane potential. Our study highlighted the pivotal role of *P. gingivalis*-LPS in the repression of serum response factor (SRF) and its co-factor p49/STRAP that regulate the actin cytoskeleton. The LPS treatment repressed the genes involved in mitochondrial function and biogenesis. *P. gingivalis*-LPS negatively altered oxidative phosphorylation and glycolysis and reduced total adenosine triphosphate (ATP) production. Additionally, it specifically altered the mitochondrial functions in complexes I, II, and IV of the mitochondrial electron transport chain. Thus, it is conceivable that *P. gingivalis*-LPS causes mitochondrial dysfunction through oxidative stress and inflammatory events in neurodegenerative diseases.

## 1. Introduction

Periodontitis is one of the most common oral chronic inflammatory diseases that is triggered by bacterial microorganisms. Increasing evidence indicates the correlation between chronic periodontitis and dementia [1,2]. One of the known periodontal bacterial species enriched in periodontitis disease is *Porphyromonas gingivalis (P. gingivalis),* an oral gram-negative anaerobe. It is one of the keystone species in the development of periodontal disease and an important factor responsible for various systemic diseases associated with aging, mainly neurodegenerative diseases, by promoting the development of Aβ plaques, cognitive impairment, perturbed motor control, and dementia [3]. *P. gingivalis* has the potential to induce neuroinflammation via its intracerebral entry or entry of its virulence factors through various direct and indirect penetration mechanisms [4,5]. It has a wide variety of virulence factors, including lipopolysaccharide (LPS), lipoteichoic acids, outer membrane vesicles (OMVs), gingipains and fimbriae [6]. LPS, a bacterial endotoxin, is a major constituent of the outer membrane of *P. gingivalis* and reaches the neuronal cells through the OMVs, where it interacts with pattern recognition receptors, such as toll-like receptors (TLR) 2 and 4 [7]. It plays a critical role in mediating inflammation and stimulating cells to secrete pro-inflammatory cytokines, mainly IL-1β, IL-6, TNF-α, NO, and reactive oxygen species (ROS) through toll-like receptor (TLR) 4 and nuclear factor-κB [8,9,10]. It is well documented that LPS derived from different bacterial species, e.g., *P. gingivalis*-LPS and *Escherichia coli*-LPS, differs in structure and functions and differently activates TLRs and regulates the production of cytokines [11,12].

*P. gingivalis*-LPS has been found in human Alzheimer’s disease (AD) brains, which suggests that *P. gingivalis*-LPS infection of the brain plays a role in AD pathogenesis [13,14,15]. There are some hallmarks of neurodegeneration and AD pathology, which are linked with oxidative stress, such as neuroinflammation and ROS production, that contribute to disease progression [16]. In AD, phospho-tau and Aβ negatively affect the neuronal cells by compromising the energy supply and altering mitochondrial functions [17]. Mitochondria are well-known cellular organelles that are mainly involved in the production of cellular energy (adenosine triphosphate; ATP), balancing ROS, and mediating cell death pathways [18,19,20]. The imbalance in mitochondrial function contributes to increased ROS generation and reduced ATP production, and all those circumstances also have direct and serious effects on the cells, which ultimately cause oxidative stress (OS) [21,22]. The mitochondrial inner membrane has five multienzyme complexes (Complexes I–V) that control oxidative phosphorylation (OXPHOS), and among all, complex-I, II and III have been reported as the main ROS producers [23,24,25]. Excess production of ROS also affects the lipids to produce lipid peroxidation products, including 4-hydroxynonal (4-HNE), which has also been found to be greatly increased in AD [26,27].

*P. gingivalis* and its LPS are known to alter the cellular cytoskeleton dynamics in different cell types [28,29]. Several aspects of neuronal mitochondrial dynamics might be related to the differential distribution and dynamics of cytoskeletal elements, and actin is considered to be crucial to mediate mitochondrial morphological alterations and short-range mitochondrial movements [30,31]. Actin dynamics is regulated by a serum response factor (SRF), an important transcription factor that is well known to be involved in multiple biological processes by maintaining cellular cytoskeleton and mitochondrial dynamics [30,31]. Moreover, SRF binding protein p49/STRAP has also been shown to be interacting with various mitochondrial genes, such as a subunit of complex I- NADH dehydrogenase ubiquinone oxidoreductase subunit AB1 (NDUFAB1), peroxisome proliferator-activated receptor gamma coactivator 1-alpha (PGC 1α) and mitofusin 1 and 2 thus, SRF along with p49/STRAP regulates mitochondrial function and dynamics [32,33,34].

SH-SY5Y cells express a number of dopaminergic neuronal markers in both undifferentiated and differentiated states. Although, differentiated SH-SY5Y cells are better for neuronal studies because they express mature neuronal markers than undifferentiated SH-SY5Y cells [35]. But, it has also been reported that the undifferentiated SH-SY5Y cells have some properties similar to mature neurons, and their low metabolic rate helps them to adapt their metabolism according to available substrates, whereas differentiated SH-SY5Y cells have altered mitochondrial functioning with reduced mitochondrial membrane potential and reduced expression of energetic stress response genes [36,37]. Considering the relevance of neuroinflammation and oxidative stress in a myriad of dementia-related pathologies, we sought to better understand the specific consequences of *P. gingivalis*-LPS-mediated mitochondrial dysfunction in undifferentiated SH-SY5Y cells. We also propose that understanding the mitochondrial dynamics and improving its function should be considered an important therapeutic intervention against *P. gingivalis*-LPS mediated neurodegenerative diseases.

## 2. Results

### 2.1. P. gingivalis-LPS Upregulates the Expression of Alzheimer Disease Related Dementia and Neuroinflammatory Markers

**Figure 1:**
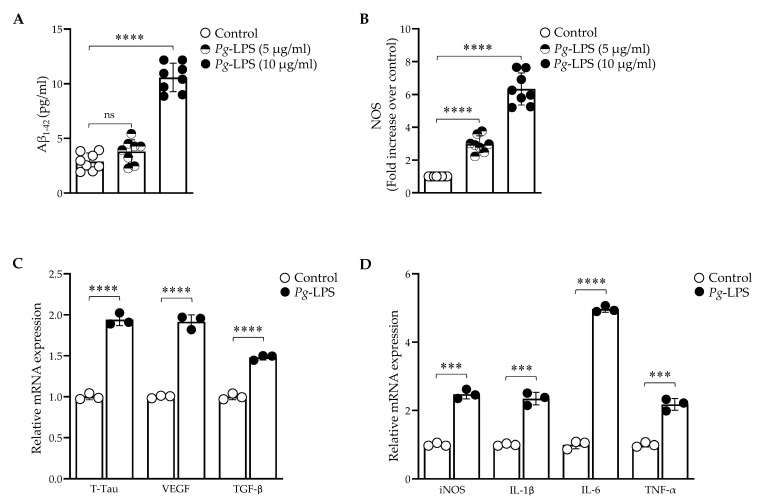
*P. gingivalis*-LPS upregulated neuroinflammatory markers associated with AD and ADRD. (**A**) Aβ_1–42_ ELISA levels were significantly increased with 10.0 μg/mL LPS treatment (*n* = 8). (**B**) Enzymatic intracellular NOS was increased with 5.0 and 10.0 μg/mL LPS concentrations (*n* = 8). The relative mRNA expression was increased with 10.0 μg/mL LPS treatment (*n* = 3): (**C**) T-Tau, VEGF, and TGF-β. (**D**) iNOS, IL-1β, IL-6, and TNF-α. *** *p* < 0.001, **** *p* < 0.0001, ns: *p* > 0.05.

The treatment of *P. gingivalis*-LPS (0–20.0 μg/mL) on SH-SY5Y cells for 24 h had no effect on the cell viability, whereas the LDH assay showed a slight increase in the cytotoxicity at 20.0 μg/mL (Supplementary Figures S1A,B). LPS treatment at 10.0 μg/mL significantly increased the Aβ_1–42_ levels as compared to the untreated control, and no change was observed at 5.0 μg/mL (Figure 1A). Thus, a 10.0 μg/mL concentration of LPS for 24 h treatment was used for further experiments.

The overproduction of nitric oxide by intracellular NO synthase was observed after the treatment (Figure 1B). The relative mRNA expression of Alzheimer’s disease-related dementia (ADRD) biomarkers, T-Tau (Total-Tau), VEGF, and TGF-β was significantly increased (Figure 1C). The neuroinflammatory markers iNOS, IL-1β, IL-6, and TNF-α were also detected at high levels (Figure 1D). Thus, *P. gingivalis*-LPS induced pathogenesis of AD and ADRD markers through neuroinflammation.

### 2.2. P. gingivalis-LPS Induces Mitochondrial ROS and Decreases Membrane Potential Mediated by TLR4

**Figure 2:**
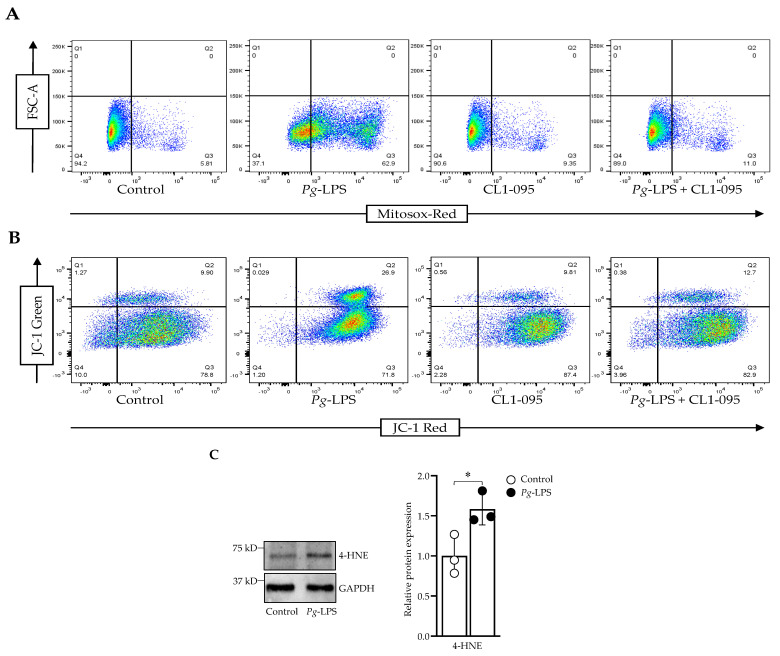
*P. gingivalis*-LPS induced oxidative stress and decreased membrane potential. (**A**) Cells were stained with MitoSOX Red, and ROS-producing cells were sorted using flow cytometry. (**B**) JC-1 staining was used to assess the decline in the membrane potential. (**C**) Western blot analysis of 4-HNE and GAPDH was used as a loading control (*n* = 3). * *p* < 0.05.

*P. gingivalis*-LPS treatment specifically activated the TLR4 mRNA expression, and its expression was recovered using the TLR4 inhibitor CLI-095 (Supplementary Figure S2). The ROS production was elevated significantly with 10.0 μg/mL LPS treatment. The treatment of CLI-095, along with the LPS, prevented ROS production (Figure 2A). With the increased ROS production, the membrane potential was significantly decreased, as indicated by the shift from red to green fluorescence with the LPS treatment (Figure 2B). CLI-095, along with the LPS, retrieved the membrane potential as well. Constitutively, 4-HNE expression was increased during the LPS treatment (Figure 2C).

### 2.3. P. gingivalis-LPS Downregulates Serum Response Factor and p49/STRAP

**Figure 3:**
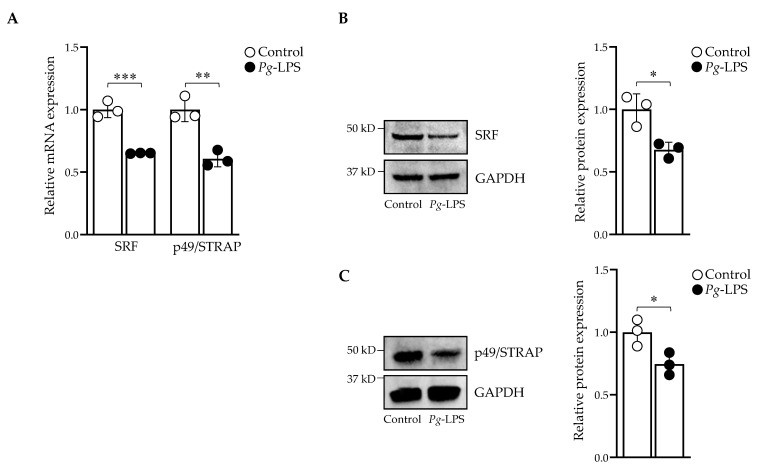
*P. gingivalis*-LPS repressed SRF and p49/STRAP. (**A**) The mRNA expression of SRF and p49/STRAP was quantified by qPCR. Western blot analysis of (**B**) SRF, and (**C**) p49/STRAP. GAPDH was used as a loading control. (*n* = 3). * *p* < 0.05, ** *p* < 0.01, *** *p* < 0.001.

*P. gingivalis*-LPS affects the actin cytoskeleton, and SRF regulates the actin [28,29]. We sought to investigate the effect of *P. gingivalis*-LPS treatment on SRF and co-factor p49/STRAP. The mRNA and protein expression levels of SRF and p49/STRAP were repressed with 10.0 μg/mL LPS (Figure 3A–C). Thus, *P. gingivalis*-LPS downregulated the expression levels of both SRF and p49/STRAP.

### 2.4. P. gingivalis-LPS Represses the Mitochondrial Biogenesis, Fission and Fusion Genes

**Figure 4:**
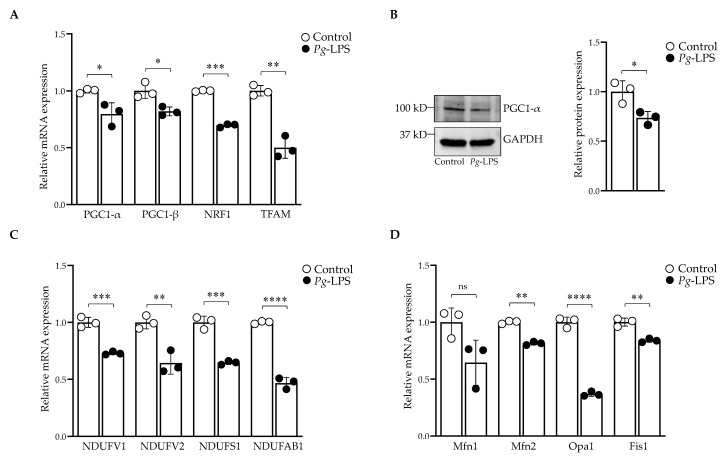
Downregulation of mitochondrial genes involved in biogenesis, fission, and fusion. (**A**) RT-qPCR analysis of *PGC-1α*, *PGC-1β, NRF1* and *TFAM* (**B**) Western blot analysis of PGC-1α. GAPDH was used as a loading control. (**C**) RT-qPCR analysis of mitochondrial complex-I genes. (**D**) *Mfn2*, *Fis1* and *Opa1* genes were downregulated (*n* = 3). * *p* < 0.05, ** *p* < 0.01, *** *p* < 0.001, **** *p* < 0.0001, ns: *p* > 0.05.

*P. gingivalis*-LPS significantly altered the mRNA expression of *PGC-1α*, *PGC-1β, NRF1* and *TFAM* (Figure 4A) as well as the protein expression of PGC-1α (Figure 4B). The LPS treatment also repressed the mitochondrial complex I subunit genes *NDUFV1*, *NDUFV2*, *NDUFS1* and *NDUFAB1* (Figure 4C). Furthermore, LPS treatment also affected the genes involved in mitochondrial fission and fusion. The mRNA expression level of mitofusin-2 (*Mfn2*), mitochondrial fission-1 (*Fis1*) and optic atrophy-1 (*Opa1*) were repressed, whereas the *P. gingivalis*-LPS-induced decrease of mitofusin-1 (*Mfn1*) did not reach statistical significance for three repetitions (Figure 4D).

### 2.5. P. gingivalis-LPS Alters Oxidative Phosphorylation, Glycolysis and Reduces the ATP

**Figure 5:**
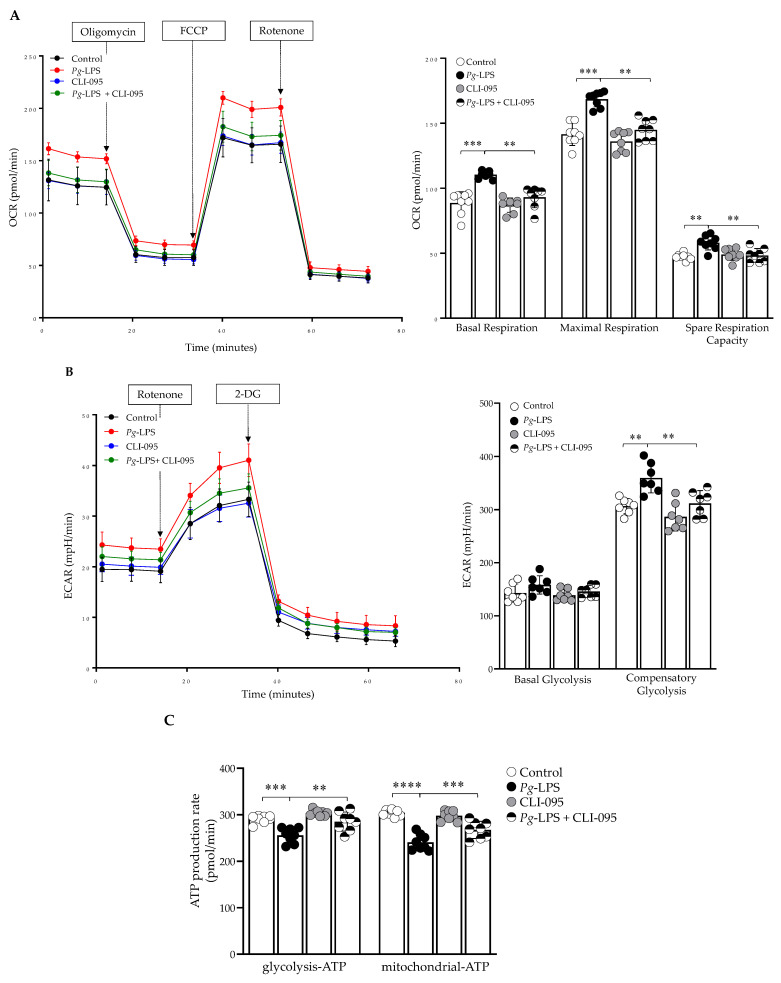
Mitochondrial functional analysis of OCR, ECAR and total ATP. (**A**) The basal respiration, maximum respiration, and spare respiratory capacity were increased with the treatment. (**B**) ECAR, basal and compensatory glycolysis were determined (**C**) Total ATP production was reduced with the treatment. CLI-095 (1 μM) was used to recover the OCR, ECAR, and ATP (*n* = 8). ** *p* < 0.01, *** *p* < 0.001, **** *p* < 0.0001.

The LPS treatment significantly altered the parameters of mitochondrial function, as evidenced by increased oxygen consumption rate (OCR), which resulted in increased basal respiration, maximal respiratory capacity, and spare respiratory capacity (Figure 5A). In the glycolytic pathway, basal glycolysis was not affected, but the compensatory glycolysis was increased with the treatment (Figure 5B). In addition, the ATP production from OCR and glycolysis (ECAR) was reduced with the LPS treatment (Figure 5C). CLI-095, along with *P. gingivalis*-LPS treatment, significantly improved OCR, ECAR, and ATP production, which demonstrated TLR4-dependent LPS function.

### 2.6. P. gingivalis-LPS Specifically Alters the Mitochondrial Function in Complex I, II, and IV

**Figure 6:**
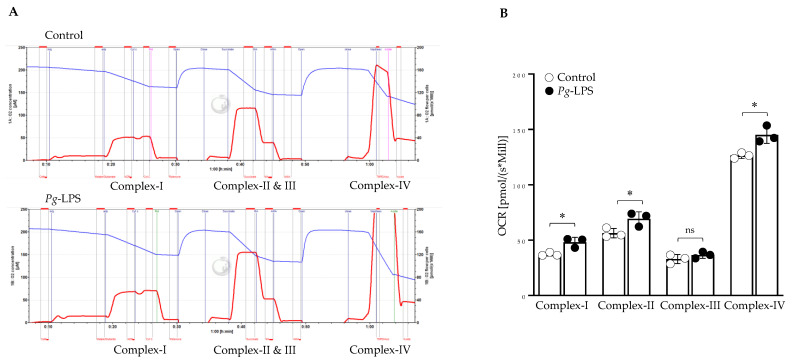
High-resolution respiratory analysis. (**A**,**B**) LPS at 10.0 µg/mL for 24 h was used for the treatment. Cells were permeabilized with digitonin and utilized to determine the OCR level at complexes of ETC (*n* = 3). * *p* < 0.05, ns: *p* > 0.05.

High-resolution respiratory analysis using Oxygraph O2k in the intact SH-SY5Y cells treated with LPS confirms the dysfunction of mitochondrial OCR (Supplementary Figure S3). With the substrate-inhibitor titration, we specifically targeted the different complexes of the Electron transport chain (ETC). The LPS-treated cells had increased respiration rates in complex I, II and IV, but complex III was unaffected (Figure 6).

### 2.7. TLR4 Expression, Actin Assembly and Mitochondrial Morphology

**Figure 7:**
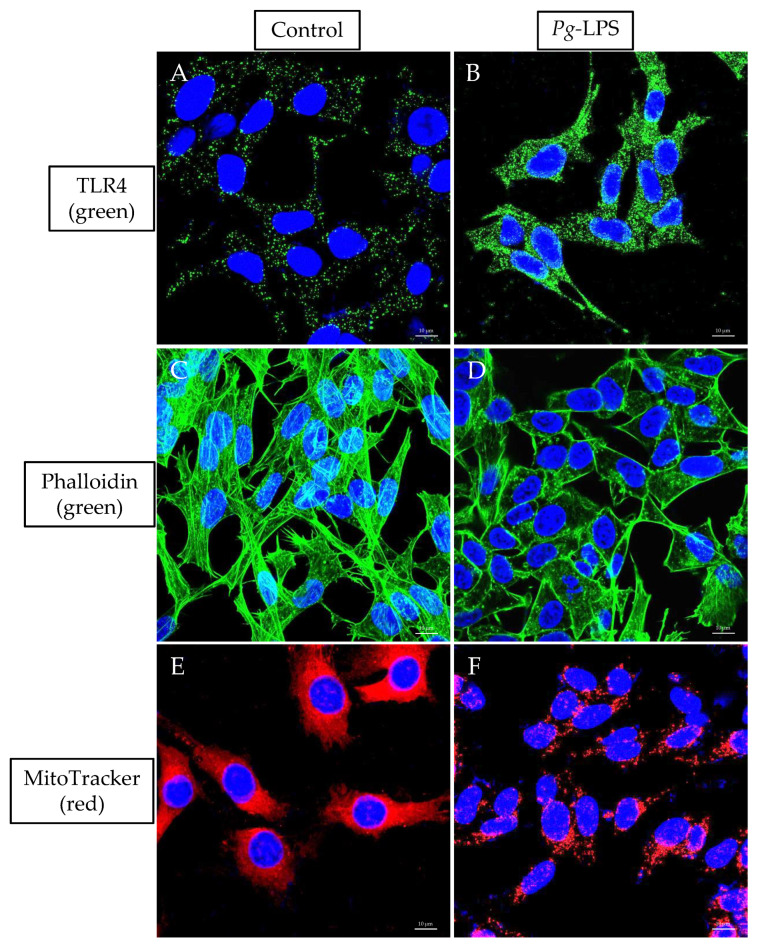
Immunolabeling of TLR4, Actin and MitoTracker. The cells were incubated with Anti-TLR4 Antibody (green), phalloidin (green) and MitoTracker (red) conjugated dyes. DAPI (blue) was used for nuclear counterstaining. Untreated (**A**,**C**,**E**) and LPS-treated (**B**,**D**,**F**). A 63× oil objective was used; scale bars indicate 10 μm.

LPS treatment activated the TLR4 expression (Figure 7B) compared to the untreated cells (Figure 7A). In f-actin phalloidin staining, the actin filaments were altered with 10.0 μg/mL LPS treated cells (Figure 7D), whereas the actin filaments were arranged intact in the untreated cells (Figure 7C). Furthermore, *P. gingivalis*-LPS treatment reduces the mitochondrial mass in the MitoTracker staining (Figure 7F) as compared to untreated control cells (Figure 7E).

## 3. Discussion

Recent research has revealed the important correlation between mitochondrial dysfunction in the pathophysiology of neurodegenerative diseases [38,39]. The role of mitochondria during neuroinflammation and neurodegeneration has unraveled mitochondria-related immunometabolic processes that may serve as promising therapeutic targets for AD and ADRD [40,41]. *P. gingivalis*-LPS can induce neuroinflammation and lead to the progression of neuropathological changes. However, the definite mechanisms of *P. gingivalis*-LPS-mediated mitochondrial dysfunction remain under-explored. Our study utilized undifferentiated SH-SY5Y cells to explore the hypothesis that LPS-mediated mitochondrial dysfunction could be the origin of oxidative stress in neurodegenerative diseases.

*P. gingivalis*-mediated inflammasome activity and neuroinflammation have been shown to be activated in AD brains [42,43]. Herein, we demonstrated the increased expression of soluble *A*β_1–42_ peptide, T-Tau protein, VEGF and TGF-β after *P. gingivalis*-LPS treatment. It also upregulated the expression of several neuroinflammatory markers such as intracellular NOS, iNOS, IL-1β, IL-6 and TNF-α (Figure 1). These observations validate the potential role of *P. gingivalis*-LPS in amyloidogenesis, tauopathy and in neuroinflammation. Next, we tested whether TLR2 or TLR4 or both were activated with *P. gingivalis*-LPS treatment. Our findings indicated that *P. gingivalis*-LPS acted exclusively through TLR4. (Supplementary Figure S2 and Figure 7A,B). Others have also shown that LPS-induced neuroinflammation is mediated by the activation of the TLR4 signaling pathway [44,45]. In several studies, it has been reported that neurons can express both TLR2 and TLR4, indicating a critical role of these receptors in neuroinflammatory responses [46,47]. Oxidative stress induces neuroinflammation and neurodegeneration [48]. The identification of *P. gingivalis*-LPS-induced ROS accumulation, reduced MMP, and elevated protein expression of 4-HNE in neuroblastoma cells underscores an important finding (Figure 2). Moreover, it has been reported that LPS from *P. gingivalis* increases oxidative stress in periodontal ligament fibroblasts [49] as well as in brain endothelial cells [50].

*P. gingivalis* and its LPS regulate cellular cytoskeleton dynamics in different cell types [28,29]. We sought to investigate the effects of *P. gingivalis*-LPS on the transcriptional activity of SRF and its co-factor p49/STRAP. SRF is a dispensable transcription factor for cellular growth, maintaining the cellular cytoskeleton, and it mediates mitochondrial function [30,31]. Interestingly, we found that *P. gingivalis*-LPS repressed the expression of both SRF and co-factor p49/STRAP (Figure 3), suggesting its role in altered actin morphology and mitochondrial dynamics (Figure 7C,D). Others have also shown that SRF regulates the actin cytoskeleton [51].

PGC1-*α* and PGC1-β are transcriptional coactivators and serve as the main regulators of mitochondrial biogenesis and function [52]. Several transcription factors, including NRF-1 and 2, TFAM, are activated by PGC1-*α* to increase the transcription of genes related to mitochondrial biogenesis and function [53]. Our study showed that *P. gingivalis*-LPS downregulated the expression of *PGC1-α, PGC1-β, NRF-1* and *TFAM*, and mitochondrial fission and fusion genes and repressed the gene expression of complex-I genes (Figure 4). The downregulation of PGC1-*α* has been reported in various inflammatory conditions [53] and negatively regulated the genes involved in mitochondrial biogenesis and function [54].

Next, we investigated the functional aspects of mitochondrial oxidative phosphorylation and glycolysis. The OCR was significantly increased with *P. gingivalis*-LPS treatment (Figure 5A). It has been reported that *P. gingivalis*-LPS treatment significantly increased respiration rates because of oxidative stress in human gingival fibroblast cells [55]. This study demonstrated that with LPS treatment, H_2_O_2_ production was enhanced, and ATP generation was reduced, which has been linked to increased oxygen demand through increased mitochondrial respiration (55). In glycolytic parameters, basal glycolysis was unaffected, while compensatory glycolysis was increased (Figure 5B). Furthermore, total ATP production (glyco-ATP and mito-ATP) was greatly reduced with LPS treatment (Figure 5C). Others have also reported reduced ATP production in both human gingival fibroblast and endothelial cells after *P. gingivalis* and its LPS treatment [55,56]. In addition, *P. gingivalis*-LPS significantly altered the mitochondrial respiration in complex-I, II, and IV but did not affect complex-III of the electron transport chain (Figure 6). Complex-I, II, and III have been reported as major producers of significant amounts of ROS [23,24,25], but our findings suggest that complex-I and II might be responsible for producing ROS and thereby inducing oxidative stress and mitochondrial dysfunction. It has been demonstrated that mitochondrial dysfunction has been linked to the increased oxygen demand due to increased mitochondrial respiration, which tilts the balance towards preferential ROS production instead of ATP [55] and hence worsens neuroinflammation.

In conclusion, our study has provided evidence that *P. gingivalis*-LPS triggered oxidative stress resulting in mitochondrial dysfunction and neuroinflammation in SH-SY5Y cells. Interestingly, TLR4-specific inhibitor CLI-095 recovered the LPS-induced mitochondrial dysfunction (Figure 2 and Figure 5). CLI-095 has been tested clinically for antimetastatic effects [57]. Our results suggest that CLI-095 could potentially be useful as a therapeutic agent against *P. gingivalis*-LPS-mediated neuroinflammation, oxidative stress and mitochondrial dysfunction in AD and ADRD. However, future studies are required to completely understand the mechanisms by which *P. gingivalis*-LPS would reach the cytosol of the stimulated cells to produce injury and whether CLI-095 could inhibit its adverse effects in-vivo.

## 4. Materials and Methods

### 4.1. Cell Culture, Cell Viability and LDH Assay

The SH-SY5Y cell line and all the cell culture reagents were previously described [58]. The ultrapure *P. gingivalis*-LPS and CLI-095 were obtained from InvivoGen, San Diego, CA, USA, and used as per the manufacturer’s instructions. Cell viability towards *P. gingivalis*-LPS was determined by using an MTS assay as described [54]. CytoTox 96^®^ cytotoxicity assay was used to quantify the lactate dehydrogenase released in the cell culture supernatant after 24 h of *P. gingivalis*-LPS treatment (CytoTox 96^®^, Promega, Madison, WI, USA). The absorbance at 490 nm was measured with the microplate reader (BioTek Synergy H1).

### 4.2. Quantification of Aβ_1–42_, NOS and Reverse-Transcriptase qPCR

The quantitative analysis of Aβ_1–42_ was determined by using a human amyloid beta (aa1-42) quantikine ELISA kit, as per the manufacturer’s instructions (R&D Systems). Intracellular NOS activity was determined using a commercially available kit per the manufacturer’s instructions (Intracellular NOS Assay Kit, Abcam, Cambridge, UK, ab211085). The fluorescence intensity was determined at Ex/Em = 485/530 nm (BioTek Synergy H1). RT-qPCR was performed as previously described [59]. The primer sequences used to quantify the gene expression are described in Supplementary Table S1.

### 4.3. Flow Cytometry

The mtROS levels were quantified by MitoSOX Red (ThermoFisher, Eugene, OR, USA; M36008). Briefly, SH-SY5Y cells were seeded at a density of 1 × 10^6^ cells/well in a 6-well plate and treated with 10.0 μg/mL concentration of *P. gingivalis*-LPS for 24 h. CLI-095 (1 μM) was added to the cells 1 h before treatment, used alone and in combination with *P. gingivalis*-LPS treatment [60]. After 24 h, cells were stained with 5 µM MitoSOX Red for 10 min. JC-1 fluorescent dye (5 µg/mL), an indicator of mitochondrial membrane potential (MMP), was used to stain the cells for 10 min (ThermoFisher, Eugene, OR, USA; T3168). The cells were centrifuged and washed gently three times with warm HBSS buffer, followed immediately by flow cytometry analysis. The fluorescence intensity was quantified by a flow cytometer (BD LSRFortessa^TM^, Franklin Lakes, NJ, USA) and analyzed by FlowJo_v10.8.1 software.

### 4.4. Western Blot

Western blots were performed as previously described [30]. The primary antibodies used were PGC-1α (ab106814), SRF (G-20, SC-335), p49/STRAP [31] and anti-4HNE antibody (ab46545). The secondary antibodies used were anti-mouse HRP (Invitrogen, Carlsbad, CA, USA; 62-6520), anti-goat HRP (Santa Cruz, Dallas, TX, USA; sc-20200), and anti-rabbit AP (Bio-Rad, Hercules, CA, USA; 64251130). iBright™ CL1500 (Invitrogen) was used for imaging. Protein expression was quantified using ImageJ software v1.53t (National Institutes of Health, Bethesda, MD, USA).

### 4.5. Mitochondrial Oxygen Consumption Rate, Glycolysis and ATP Production

SH-SY5Y cells were seeded at a density of 5 × 10^4^ cells/well in XFe96 Well plates (Seahorse Bioscience, Billerica, MA, USA). The cells were treated with a 10.0 μg/mL concentration of *P. gingivalis*-LPS for 24 h. After treatment, cells were subjected to extracellular flux analysis using the XF Cell Mito Stress Test Kit (Agilent, Santa Clara, CA, USA; 103015-100), XF Glycolytic Rate Assay Kit (Agilent, Santa Clara, CA, USA; 103344-100), and XF Real-Time ATP Rate Assay (Agilent, Santa Clara, CA, USA; 103592-100) respectively.

### 4.6. High-Resolution Respirometry

The Oxygraph 2k (O2K) respirometer (Oroboros Instruments GmbH, Innsbruck, Austria) was utilized to examine the mitochondrial function in the intact cells (2.5 × 10^6^/sample), as previously described [54,61]. Furthermore, the OCR in the complexes was measured according to the substrate-inhibitor-titration protocol as described [54]. Briefly, 5 × 10^6^ cells were incubated with digitonin (Sigma, Milwaukee, WI, USA; D5628; 8 μM/million cells) and prepared in MiRO5 buffer for 20 min at 4 °C to permeabilize the cells. Data analysis was performed with DatLab 6.2 software (Innsbruck, Austria), and the OCR of intact cells and from each individual mitochondrial complex was expressed as oxygen flux (pmol/s*Million Cells).

### 4.7. Immunofluorescence

SH-SY5Y cells were seeded with 1.0 × 10^6^ cells per well in a 35 mm MatTek microscopy glass dish (Fisher Scientific, Waltham, MA, USA; PDCFOS30) and treated with 10.0 μg/mL LPS for 24 h. As described previously, cells were fixed, permeated, and blocked first [54]. The cells were stained with MitoTracker™ Red and ActinGreen, as described in [54]. TLR4 staining was done by incubating the cells with Anti-TLR4 conjugated Antibody (1:100) Alexa Fluor^®^ 488 (Santa Cruz, Dallas, TX, USA; sc-13593 AF488, HTA125) overnight at 4 °C as per manufacturer’s instructions. Cells were then counterstained for 5 min with DAPI (1:1000) to label the nuclei (ThermoFisher, Waltham, MA, USA; D1306). The fluorescent images were captured by using a Zeiss LSM 880 confocal microscope (Carl Zeiss Microscopy, White Plains, NY, USA). The collected images were processed with ZEN blue 3.2 software (Carl Zeiss Microscopy, White Plains, NY, USA).

### 4.8. Quantification and Statistical Analysis

Statistical analysis was carried out using GraphPad Prism 9.1.1 Software Inc. (Dotmatics, Boston, MA, USA). Data represent mean values ± SD of at least 3 independent experiments, or as indicated by n. The 2-tailed Student’s *t*-test was used to determine the statistical significance between 2 samples originating from the same population, and the statistical significance between multiple groups was determined by 1-way ANOVA followed by Tukey’s multiple comparison test (* *p* < 0.05; ** *p* <0.01; *** *p* < 0.001, **** *p* < 0.0001, ns: non-significant).

## Data Availability

The raw data are available without reservation upon reasonable request.

## Supplementary Materials

The supporting information can be downloaded at: https://www.mdpi.com/article/10.3390/ijms24020950/s1.
