# Supplementary material for: P. gingivalis-LPS Induces Mitochondrial Dysfunction Mediated by Neuroinflammation through Oxidative Stress
Source: Int J Mol Sci. 2023 Jan 4;24(2):950. doi: 10.3390/ijms24020950 (PMC9861869; doi:10.3390/ijms24020950)
Supplement: Supplementary file 1 [file ijms-24-00950-s001.zip › ijms-2101880-supplementary.pdf]

Supplementary Data

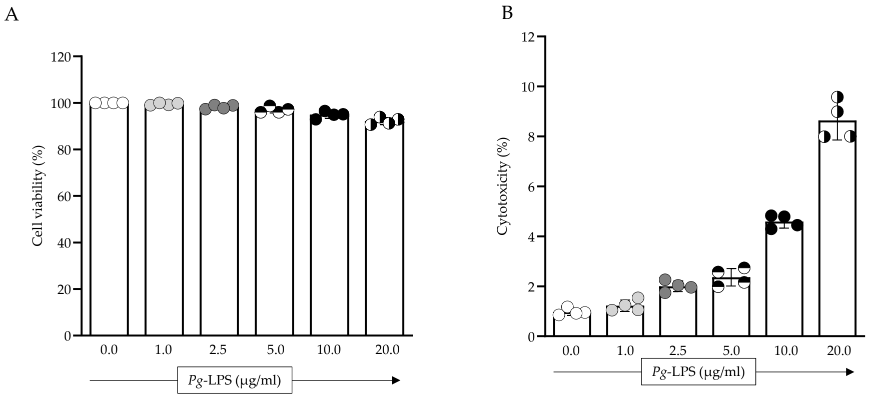

Supplementary Figure S1. Effect of *P. gingivalis*-LPS on cells viability with MTS assay (A) and cytotoxicity with LDH assay (B) in SH-SY5Y cells treated with 0, 1.0, 2.5, 5.0, 10.0 and 20.0 μg/ml concentrations of *P. gingivalis*-LPS for 24 hours and untreated cells were used as control.

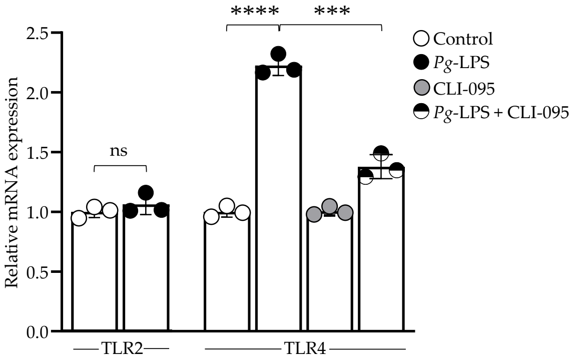

Supplementary Figure S2. Effects of *P. gingivalis*-LPS on mRNA expression of TLR2 and TLR4. RT-qPCR analysis showing the gene expression of TLR2 in untreated control and cells treated with 10 μg/ml concentration of *P. gingivalis*-LPS, and TLR4 gene expression in untreated control, cells treated with 10 μg/ml concentration of *P. gingivalis*-LPS, CLI-095 (1μM), *P. gingivalis*-LPS + CLI-095 (1μM) (n=3). ***$p < 0.001$, ****$p < 0.0001$, ns: $p > 0.05$.

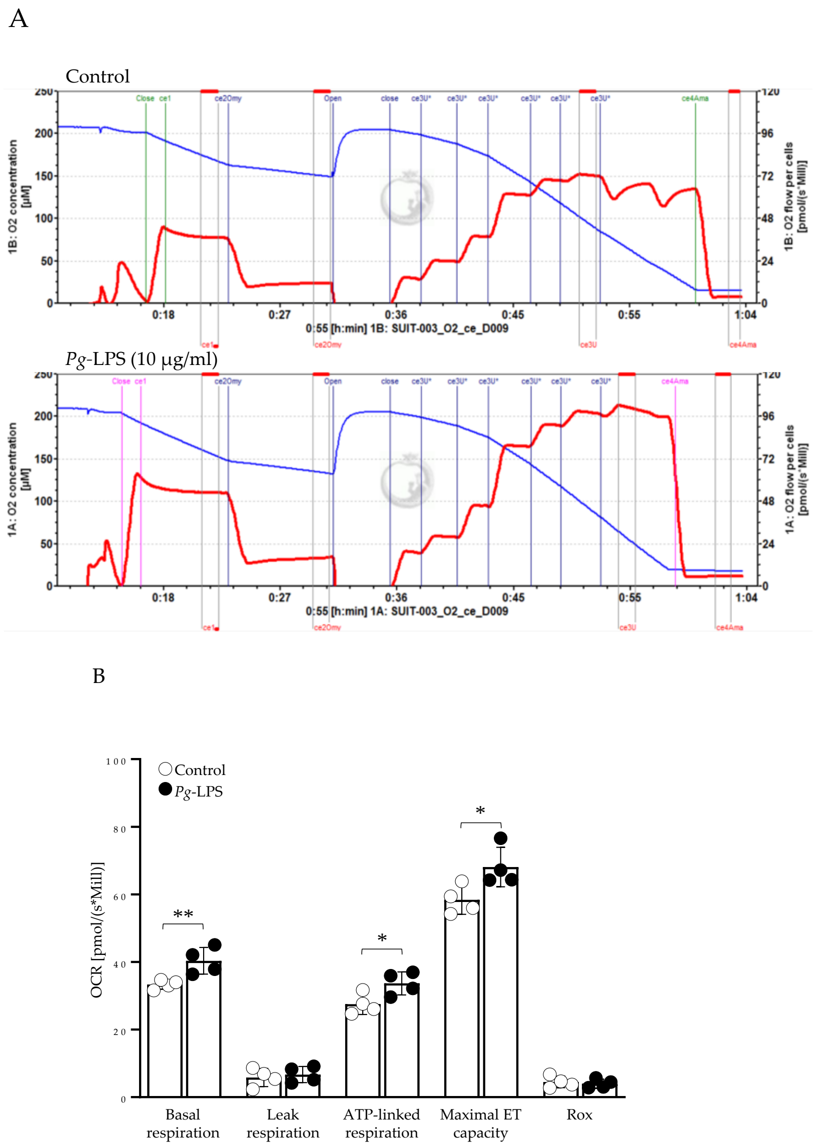

Supplementary Figure S3. High-resolution respiratory analysis of oxidative phosphorylation in Oroboros O2K respirometer. (A) OCR and (B) Basal respiration, leak respiration, ATP-linked respiration, Maximum ET capacity was measured from intact SH-SY5Y cells treated with 10 µg/ml *P. gingivalis* LPS for 24 hours and untreated cells were used as control (n=4). *$p < 0.05$, **$p < 0.01$, ns: $p > 0.05$.

Supplement Table S1. List of primer sequences used for qPCR analysis.

Primer Name	Primer Sequence (5'--3')
Tau-F	5′-GATTGGGTCCCTGGACAATA-3′
Tau-R	5′-GTGGTCTGTCTTGGCTTTGG-3′
VEGF-F	5′- TGCAGATTATGCGGATCAAAC C-3′
VEGF-R	5′-TGCATTCACATTTGTTGTGCTGTAC-3′
TGF-β-F	5′- GCGTGCTAATGGTGGAAACC-3′

TGF-β-R	5′- CGGAGCTCTGATGTGTTGAAGA-3′
IL-1β-F	5′-ATGGGATAACGAGGCTTATGTG-3′
IL-1β-R	5′-CAAGGCCACAGGTATTTTGTC-3′
*IL-6*F	5′-ACTTGCCTGGTGAAAATCAT-3′
*IL-6*R	5′-CAGGAACTGGATCAGGACTT-3′
TNF-α-F	5′-TCAGCAAGGACAGCAGAGG-3′
TNF-α-R	5′-CAGTATGTGAGAGGAAGAGAACC-3′
iNOS-F	5′-GCAGAATGTGACCATCATGG-3′
iNOS-R	5′-ACAACCTTGGTGTTGAAGGC-3'
TLR2-F	5'- CTTTCACTGCTTTCAACTGGTA -3'
TLR2-R	5'-TTGCGGTCACAAGACAGAG -3'
TLR4-F	5'-GACTTGCGGGTTCTACATCA-3'
TLR4-R	5'-GAGGTGGCTTAGGCTCTGATA-3'
SRF-F	5'- GGGCCGCGTGAAGATCAA-3'
SRF-R	5'- GTCAGCGTGGACAGCTCATA-3'
p49/STRAP-F	5'- GAAGACACCTGCTGACCCAA-3'
p49/STRAP-R	5'- TCTTCTCCGCCATCACTGTT-3'
PGC-1α-F	5'- ACCCACAGAGAACAGAAACAG-3'
PGC-1α-R	5'- GGGTCAGAGGAAGAGATAAAGTTG-3'
PGC-1β –F	5'- ACTACTTCGCTGACACGCAG-3'
PGC-1β –R	5'- CTCTGAGTTCTCTGGGCACC-3'
NRF1-F	5'- GGAAACGGCCTCATGTATTTG-3'
NRF1-R	5'- GTTTGGAGGGTGAGATACAGAG-3'
TFam-F	5'- TTTCTCCGAAGCATGTGGG-3'
TFam-R	5'-GCCAAGACAGATGAAAACCAC -3'
MFN1-F	5'- TGGCATCTGTGGCCGAGTT-3'
MFN1-R	5'-GAAACAGGTTCTGCCATTATGCT -3'
MFN2-F	5'- CGCGCTTATCCACTTCCCTC-3'
MFN2-R	5'- AGAAGAGCAGGGACATTGCG-3'
Fis1-F	5'- TGACATCCGTAAAGGCATCG-3'
Fis1-R	5'-CTTCTCGTATTCCTTGAGCCG -3'
Opa1-F	5'- GGAGAACCATATTCGTTTTGACC-3'
Opa1-R	5'- AGAGCTGTTCCCTTTTCCTG-3'
NDUFV1-F	5'- CGGGTATCTGTGCGTTTCAG-3'
NDUFV1-R	5'- GGTCTTCATCCTTCAGCGAG-3'
NDUFV2-F	5'- GGCAAAATCCCAAAACCAGG-3'
NDUFV2-R	5'- TGCTTGTACACCAAATCGAGG-3'
NDUFAB1-F	5'-TCCAGGACCGTGTTCTTTACG -3'
NDUFAB1-R	5'- GGTCCAAACTGTCTAAGCCC-3'
NDUFS1-F	5'- ACTCTGACACCTTATGCACTG-3'
NDUFS1-R	5'- AACATCTGGCTCTTCCACAC-3'

F: forward primer; R: reverse primer.
